# Ecological niche conservatism spurs diversification in response to climate change

**DOI:** 10.1038/s41559-024-02344-5

**Published:** 2024-02-19

**Authors:** Huijie Qiao, A. Townsend Peterson, Corinne E. Myers, Qinmin Yang, Erin E. Saupe

**Affiliations:** 1 Key Laboratory of Animal Ecology and Conservation Biology, Institute of Zoology, Chinese Academy of Sciences, Beijing, China; 2 Biodiversity Institute, University of Kansas, Lawrence, KS USA; 3 Department of Earth and Planetary Sciences, University of New Mexico, Albuquerque, NM USA; 4 State Key Laboratory of Industrial Control Technology, College of Control Science and Engineering, Zhejiang University, Hangzhou, China; 5 Department of Earth Sciences, University of Oxford, Oxford, UK

**Keywords:** Biogeography, Ecological modelling, Evolutionary ecology, Macroecology

## Abstract

Lengthy debate has surrounded the theoretical and empirical science of whether climatic niche evolution is related to increased or decreased rates of biological diversification. Because species can persist for thousands to millions of years, these questions cross broad scales of time and space. Thus, short-term experiments may not provide comprehensive understanding of the system, leading to the emergence of contrasting opinions: niche evolution may increase diversity by allowing species to explore and colonize new geographic areas across which they could speciate; or, niche conservatism might augment biodiversity by supporting isolation of populations that may then undergo allopatric speciation. Here, we use a simulation approach to test how biological diversification responds to different rates and modes of niche evolution. We find that niche conservatism promotes biological diversification, whereas labile niches—whether adapting to the conditions available or changing randomly—generally led to slower diversification rates. These novel results provide a framework for understanding how Earth–life interactions produced such a diverse biota.

## Abstract (web summary)

The authors use a simulation framework to assess how the dynamics of species’ diversification changed with ecological niche shifts under historical climate conditions. Modelling scenarios with niche conservatism resulted in higher rates of net diversification, recapitulating empirical biodiversity patterns.

## Main

Rates of biological diversification vary dramatically across clades, regions and time^1–4^. Understanding what factors control this variation is a fundamental question in evolutionary biology. One suite of factors posited to regulate diversification are species’ climatic tolerances (or abiotic niches)^5,6^, defined as the environmental conditions allowing species to maintain viable populations^7,8^. The climatic niche determines, at least in part, the regions occupied by species over space and time, and how they respond to environmental change. Climatic tolerances may therefore affect diversification by regulating speciation or extinction processes^9,10^. Previous research has suggested that high^9,11–20^ or low^10,21–25^ rates of climate niche evolution underlie rapid species’ diversification. These opposing results point to an outstanding question that is central to understanding the sources and drivers of biological diversification.

Climate niche lability may increase rates of diversification by reducing extinction or elevating speciation rates. Rates of climate-driven extinction are expected to be low in species that evolve climatic tolerances rapidly, given their ability to adapt to and withstand novel conditions^26^. Conversely, rates of speciation may be high in species that adapt quickly to local climate conditions^27^. Populations experiencing different climate conditions over time may undergo divergent selection, leading to reproductive isolation and speciation^9,28,29^. Greater lability in climatic niches may also facilitate colonization of novel environments, creating further opportunities for allopatric speciation^10,30^. Although climate niche lability is a central tenet of these explanations, some degree of niche conservatism is still required: reproductive isolation would be difficult to maintain if species adapted fully to changing conditions over short timescales^31^.

In contrast to the hypothesis that niche lability spurs diversification, diversification may be elevated when climatic niche evolution is constrained^23–25^. Slow rates of climate niche evolution may increase diversification via elevated allopatry in geographic distributions, even as they are expected to intensify climate-driven extinction risk, which would slow diversification. Although climate is but one driver of allopatry, differences in climate across geographic space can create unsuitable regions that can isolate once-contiguous populations and interrupt gene flow. These barriers are likely to be maintained if populations are unable to adapt to the intervening unsuitable conditions^10^. Reproductive isolation can manifest via several mechanisms, including divergent adaptations to similar environmental conditions, pleiotropic consequences arising from divergence in traits unrelated to climate and epistatic interactions among genes. Ecological niche divergence is not a necessary prerequisite for fixation of these genes in separate populations, as reproductive isolation also can be facilitated by genetic drift^10^.

Thus, both the niche conservatism and niche lability hypotheses propose direct links between rates of climate niche evolution and rates of diversification, yet with contrasting expectations. Here, we use a spatially explicit mechanistic model to examine in silico^32–34^ the relationship between climate niche evolution and net diversification rate for terrestrial simulated species. We decompose the relative contributions of speciation and extinction to net diversification under varying degrees of climate niche evolution, allowing a test of the two opposing hypotheses.

## Results

**Fig. 1:**
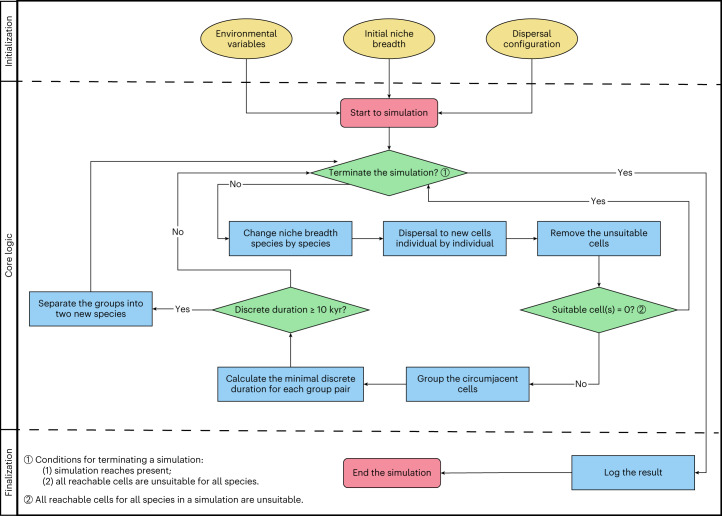
The simulation workflow. The framework and steps involved in the simulation, from initialization through to finalization.

**Fig. 2:**
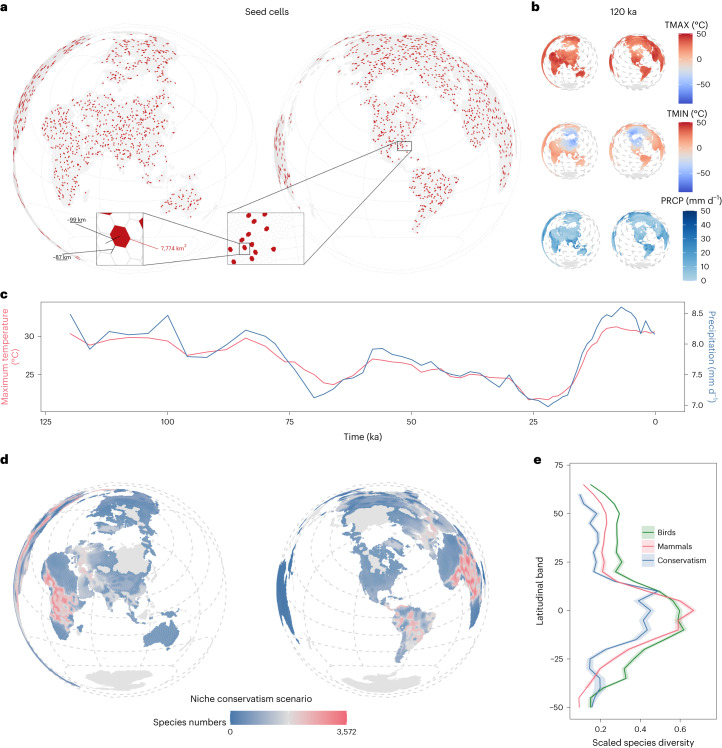
Initial seed cells, mean climate dynamics and simulation output. **a**, Initial starting points, or seed cells, for each of the simulations (*n* = 870). **b**, Climatic parameters used in the simulations. Maximum monthly temperature (TMAX), minimum monthly temperature (TMIN) and annual precipitation (PRCP) are shown for conditions 120 ka. **c**, Globally averaged maximum monthly temperature and annual precipitation dynamics over the past 120 kyr, used in the simulations. **d**, Patterns of species richness produced by the niche conservatism simulations, across all niche breadth and dispersal combinations. For richness maps produced by the other niche evolution scenarios, see Supplementary Fig. 5. Map polygons derive from the rnaturalearth R package^68^. **e**, The latitudinal diversity gradient produced at the end of the niche conservatism scenario, across all niche breadth and dispersal combinations, compared with the empirical diversity gradients for birds and mammals. Richness was calculated using the bootstrap subsampling approach described in Methods. The shading represents 95% confidence intervals, because standard deviations are large and make visualization difficult.

**Fig. 3:**
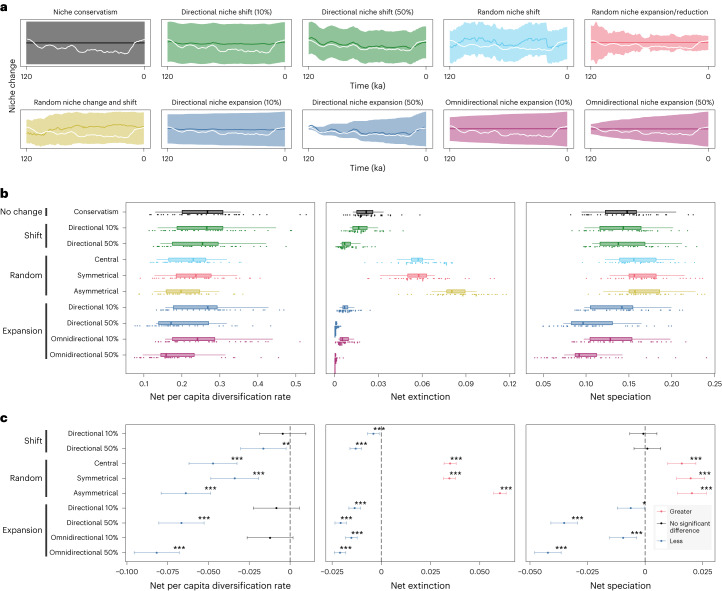
Comparison of diversification dynamics under different niche evolution scenarios. **a**, Graphical illustration of the ten niche evolution scenarios. The white line represents hypothetical climate change, the *x* axis represents time and the *y* axis represents the parameters of the niche: the dark line is the hypothetical niche centre and the shading is the hypothetical niche breadth. **b**, Mean net per capita diversification, net per capita extinction and net per capita speciation for each time window (*n* = 10) by niche breadth (*n* = 2) and dispersal scenario (*n* = 2), for a total of 40 data points in each box plot. Box plots show the minimum, first quartile, median, third quartile and maximum rate values. **c**, Results from post-hoc Tukey’s HSD, testing whether significantly different net per capita diversification, net per capita extinction and net per capita speciation rates were found in the niche conservatism scenario compared with the other niche evolution scenarios. The 95% confidence intervals show the range of possible values for the difference in means between the niche conservatism scenario and all other niche evolution scenarios. Blue indicates lower rates for the niche evolution scenarios compared with the niche conservatism scenario. Statistical significance at ***P < 0.001, **P < 0.01 and *P < 0.05. The number of data points included in each comparison is as follows: 24,177 for niche conservatism; 24,692 for directional niche shift 10%; 26,477 for directional niche shift 50%; 25,233 for directional niche expansion 10%; 27,100 directional niche expansion 50%; 25,421 for omnidirectional niche expansion 10%; 27,230 for omnidirectional niche expansion 50%; 21,482 for random niche shift; 21,653 for random niche expansion/reduction; and 19,353 for random niche change and shift. Note the ANOVA test assumes independence of the data, which is not necessarily valid when evolutionary rates are measured across multiple time bins but raw results show similar patterns (see Supplementary Figs. 4 and 9).

The simulation framework relied on the cellular automaton model of refs. ^35^ and ^22^ (Fig. 1) but differs in (1) being based on an icosahedral spherical geodesic grid that permits global dispersal and range-extension phenomena on an approximate equal-area and equidistant plane simultaneously (Fig. 2a); and (2) incorporating evolutionary change scenarios, such that the climatic niches of the simulated lineages are able to change and adapt to the environment manifested across the landscapes where they are distributed (Fig. 3a, and Supplementary Figs. 1 and 2).

Patterns of speciation and extinction were simulated in response to a dynamic climate approximated over the past 120 kyr, derived from the coupled atmosphere–ocean generalized circulation models of refs. ^36^ and ^37^ (Fig. 2b,c). Speciation occurred only allopatrically in our simulations as a direct result of geographic isolation. Simulated species could interact with spatiotemporal variation in temperature and precipitation by shifting their distributions or their climatic niches, during which process they could undergo speciation or extinction. We chose 1,000 initial simulated species locations (seed cells) randomly (Fig. 2a), which were tested under all combinations of niche breadth (two: narrow and broad), dispersal ability (two: poor and good) and niche evolution scenario (ten in total; see Fig. 3a, Supplementary Fig. 1 and Methods), resulting in 40,000 unique simulated species. Each simulated distribution started at a single cell: at the end of each simulation, the final spatial pattern of diversity was quantified, including all extant descendant species that had evolved over the course of the simulation.

Of the initial 40,000 simulations, 25,951 completed without all daughter lineages going extinct by the end of the simulation (that is, the present day). We removed 5,200 simulations (130 seed cells) prior to analysis, because these simulations were characterized by run-away speciation, resulting in run times too long (>100 hours) or requiring too much memory (>500 GB). The remaining simulations produced 685,700 species during the simulation process, of which 617,741 had non-zero ranges at the end of the simulation (Supplementary Figs. 3 and 4).

Simulations were able to replicate empirical biodiversity patterns. The distributional sizes of the simulated species under all niche evolution scenarios, aside from niche expansion, were roughly congruent with the mean range sizes of mammals and birds (Supplementary Table 2). Realistic-looking latitudinal diversity gradient and global richness patterns were also produced, as in our previous study that assessed those patterns in depth^22^. These realistic-looking patterns emerged from the niche conservatism, directional niche shift and 10% directional niche expansion scenarios, but not in the remaining niche evolution scenarios (Fig. 2d,e and Supplementary Fig. 5).

We used two statistical tests to evaluate evolutionary rates resulting from the simulations: paired *t*-tests and post-hoc Tukey’s honest significant difference tests (HSD; Supplementary Table 3). Compared with the niche conservatism scenario, net per capita diversification was significantly lower for all niche evolution scenarios except for 10% directional niche shift, 10% directional niche expansion and 10% omnidirectional expansion, for which no significant differences were found using Tukey’s HSD tests (Fig. 3b,c). Patterns were similar when examining individual niche breadth by dispersal combinations, except that more niche evolution scenarios did not differ significantly compared with the niche conservatism scenario (Supplementary Fig. 6). The paired *t*-tests produced similar results, except that only the 10% directional niche shift scenario showed no significant differences (Supplementary Table 3).

Net per capita diversification was higher in the niche conservatism scenario primarily owing to elevated rates of speciation (Fig. 3b,c, and Supplementary Figs. 3 and 4). Speciation rates were statistically elevated for the niche conservatism scenario compared with all other niche evolution scenarios, aside from the three scenarios of random change, which had significantly higher speciation rates, and the directional niche shift scenarios (10% and 50% rate), which did not differ significantly (Fig. 3b,c) using Tukey’s HSD tests. Patterns were similar when examining individual niche breadth by dispersal combinations, although more scenarios did not differ significantly (Supplementary Fig. 6). The paired *t*-tests produced congruent patterns (Supplementary Table 3).

Rates of extinction were also statistically elevated for the niche conservatism scenario compared with the other niche evolution scenarios. The exception was the three random niche evolution scenarios (Fig. 3b,c, and Supplementary Figs. 3 and 4), which had statistically higher extinction rates compared with the niche conservatism scenario. The higher extinction rates were balanced by higher speciation rates for the niche conservatism scenario, which accounts for the higher net diversification found in this scenario relative to the other niche evolution scenarios. Patterns were similar regardless of statistical test (Supplementary Table 3).

Lineages that evolved broad climatic tolerances (niche expansion scenarios) did not experience higher rates of allopatric speciation in our framework (Fig. 3b,c and Supplementary Fig. 4), contrary to the hypothesis that broad environmental tolerances may increase susceptibility to vicariance events^38^.

Elevated rates of speciation were found in the niche conservatism scenario because species under those conditions had geographic ranges that were more easily fragmented and isolated, leading to allopatry (Supplementary Fig. 7). The only niche evolution scenarios that experienced statistically greater population fragmentation were those that changed randomly; however, increased speciation under those conditions was offset by higher extinction.

## Discussion

The simulations showed clear signals regarding the hypothesized effects of climatic niche conservatism on the process of biological diversification. In short, simulations that involved evolutionary change of climatic niches either depressed rates of biological diversification or had minimal effects compared with scenarios with conserved climatic niches; in no case did the simulated evolutionary change in climatic niches lead to elevated net diversification rates. Indeed, slower rates of niche change tended to lead to increased net diversification (Supplementary Fig. 8). Thus, climatic niche conservatism often acted as a promoting factor in biological diversification in our simulation framework.

The niche conservatism scenario produced higher rates of diversification primarily owing to elevated speciation rates, rather than reduced extinction rates. Rates of speciation were elevated in the niche conservatism scenario due to species’ inability to adapt to new climatic conditions, with consequent range fragmentation and population isolation and speciation. These results are therefore conditioned on the fact that speciation predominantly occurs allopatrically. A large body of literature has addressed the question of modes of speciation and has contrasted allopatric speciation, in which divergence is driven by isolation of populations^39^, with so-called ecological speciation, in which ecological divergence among continuous or overlapping populations acts as a driving force behind population differentiation^40^. These early ideas drove development of methods for evaluating the relative frequencies of different modes of speciation^41,42^, followed by empirical testing that pointed—in most cases—to allopatric speciation mechanisms as dominant^43^.

The scenarios in which simulated species were responsive to local climate change had lower rates of extinction overall, in support of Darwin’s^44^ idea that species better able to adapt will be those that survive. However, these adaptive scenarios were often also characterized by lower speciation rates, resulting in lower rates of net diversification compared with the scenario of niche conservatism. By contrast, species simulated under scenarios of stochastically changing niches had the highest speciation rates but also the highest extinction rates, resulting in low net per capita diversification.

Previous work has found support for a positive relationship between rates of climatic niche evolution and rates of diversification in birds^13,14,19,20^, squamates^15,18^, amphibians^11,15^ and mammals^17^. Our in silico experiments point to the opposite relationship. The discrepancy between the empirical case studies and our simulated patterns may result from the shorter timescale of our simulations, which could affect how rates of biological diversification scale with rates of niche change. Alternatively, the niche change assessed in the empirical case studies may have been in the realized occupation of climates, not in changes in fundamental tolerances^5,6^, which can result in overestimation of niche lability^45,46^. It is difficult to characterize rates of climatic niche change in empirical systems, especially over evolutionary history. When uncertainty is incorporated into such analyses, rates of estimated niche evolution are often reduced^45,46^.

Our simulations involve sets of assumptions that may affect interpretation of the simulated patterns. For instance, our approach is relevant only to terrestrial species, to the niche axes of temperature and precipitation, and only reflect the relatively coarse spatial resolution of our study. Other niche dimensions and other major suites of environments over longer time intervals, especially when examined at higher spatial resolution, may show different—or even reversed—associations with net diversification. In addition, the temporal duration of our simulation (120 kyr) is relatively short on geological timescales, a constraint imposed by the paucity of temporally continuous, longer-term climate data necessary to model evolutionary dynamics. Future versions of our simulations will include longer climatic time series that are being developed, and will incorporate coastline evolution, mountain-building and other changes to the global panorama across which biological diversification takes place^47^, all of which may affect the relationship between climatic niche change and rates of net diversification. Despite these caveats, our global scale analyses have shown, across geography, environments, tolerance levels and dispersal abilities, that slower rates of climatic niche change may promote biological diversification (Supplementary Fig. 8).

Life’s occupation of nearly all existing environments on Earth demonstrates that niche evolution occurs, at least over macroevolutionary timescales. The tempo and mode of this change, however, is far from known. Changes in niches may occur most often during population fragmentation associated with the early stages of allopatric speciation^25,31,48,49^, but the timescale(s) of speciation makes this assertion difficult to test. Understanding how and when climatic niche evolution occurs is essential to predicting species’ responses to current and future environmental changes; thus, continued study of the dynamics of niche evolution is essential for effective conservation strategies.

The generality provided by these simulations allows us to address broad evolutionary questions, such as the impact of adaptation on rates of diversification^32–34^, and provides critical new insight on species-level evolutionary dynamics, even when the full complexity of the system in unknown. Here we document how niche conservatism enhances biological diversification through elevated allopatric speciation in our simulation framework. Our study therefore integrates diversification with an emerging picture of niche conservatism as a dominant pattern on relatively short timescales, which has been anticipated theoretically^50,51^ and shown empirically across taxa^49,52–54^. The results offer a mechanistic path to the evolution of rich biological diversity in many Earth systems.

## Methods

We used an eco-evolutionary simulator to model effects of climatic niche change on diversification patterns in terrestrial simulated species. The simulation framework relied on the cellular automaton model of refs. ^35^ and ^22^ (Fig. 1). Our present generation of simulations differs from our previous experiments in (1) being based on a (icosahedral) spherical geodesic grid that permits global dispersal and range-extension phenomena on an approximate equal-area and equidistant plane simultaneously (Fig. 2a); and (2) incorporating evolutionary change scenarios, such that the climatic niches of the simulated lineages are able to change and adapt to the environment manifested across the landscapes where they are distributed.

Patterns of speciation and extinction were simulated in response to a dynamic climate approximated over the past 120 kyr (Fig. 2b,c). Estimates of spatiotemporal climate change were derived from the Atmosphere-Ocean Generalised Circulation Models of refs. ^36^ and ^37^, discussed below. Simulated species could interact with spatiotemporal variation in temperature and precipitation by shifting their distributions and/or climatic niches, during which process they could undergo speciation or extinction.

Simulations were initiated under interglacial climatic conditions (Eemian, late Pleistocene, 120 kyr ago (ka)) and run forward in time to the present day using current continental configurations. Each initial lineage had the potential to go extinct or speciate, processes that were driven by changing environmental conditions. Speciation occurred when ranges were fragmented via isolation of suitable areas for at least 10 kyr, whereas extinction occurred when all occupied suitable areas were eliminated, and the species was unsuccessful at colonizing newly suitable sites. Code for the simulation is provided in our GitHub repository (https://github.com/qiaohj/ees_cpp).

### Defining simulated species

A simulated species began the simulation at a site of origin chosen randomly from across terrestrial areas globally, which were divided into 17,422 grid cells (~87 km side length, ~99 km cell spacing and 7,774 km^2^ in area) in an icosahedral spherical geodesic grid system^55^ with the dggridR v. 3.0 package in R v. 4.3 (ref. ^56^). Each grid cell in the configuration has a similar area, and each circumjacent grid cell pair has a similar distance (~99 km) at the same time.

### Defining climatic tolerances

Grid cell occupation was controlled by species’ abiotic climatic tolerances and dispersal ability in relation to the environmental conditions manifested in that cell. The temperature and precipitation values of the initial starting grid cell defined the centre of that particular simulated species’ fundamental ecological niche. Because simulations began under conditions representative of 120 ka, the simulated species could be considered as ‘warmer adapted’ in a Pleistocene-to-recent context. We applied symmetrical deviations to the values of the initial cell based on two niche breadths (narrow and broad), corresponding to temperature and precipitation tolerance ranges of 40 °C and 60 °C, and 5 mm d^−1^ and 10 mm d^−1^, respectively. Niche breadths were based on plant tolerances and represent the 20% and 80% quantiles of tolerances in the Food and Agriculture Organization of the United Nations database^57^. These basic tolerances (that is, fundamental ecological niches) were reduced by the set of environmental combinations existing and accessible to the species at a given time step, referred to as the existing ecological niche^6^. Occupation of the existing ecological niche at any given time step was reduced still further by the species’ dispersal ability.

### Defining dispersal ability

Each species was assigned a dispersal function, reflecting its ability to search outside occupied cells for other habitable cells. Dispersal in the simulation was stochastic, representing exploration, with possible colonization and range expansion, at difference from other definitions of dispersal at local scales in terms of movements of individuals. We considered two levels of dispersal ability, both defined by exponential decay curves for the probability that a species will disperse a certain number of cells. From a given occupied cell, a species was allowed to search, at maximum, four (good dispersers) or two (poor dispersers) cells in a single simulation step, approximately corresponding to distances of 400 km and 200 km, respectively. Species searched for suitable cells simultaneously from all cells currently occupied, and each occupied cell was assigned a different probability of dispersal. Species could jump over unsuitable cells to encounter suitable cells that were more spatially remote, so dispersal could occur at least occasionally between continents. Dispersal values (Supplementary Table 1) were based loosely on known dispersal of empirically derived seed-dispersal capacities in plants^58,59^.

### Climatic conditions

Climatic attributes of individual cells fluctuated, producing conditions favourable or unfavourable for a species at a given time interval, depending on whether the cell value fell within the species’ climatic niche. Dynamic climate change trajectories over the past 120 kyr were derived from transient climate simulations using state-of-the-art, coupled atmosphere–ocean–vegetation models (HadCM3) developed at the Hadley Centre, detailed in refs. ^37^ and ^36^. Three environmental parameters constrained species’ tolerances: mean monthly maximum temperature, mean monthly minimum temperature and mean monthly maximum precipitation (minimum monthly precipitation is 0 for most localities on Earth and was therefore not included).

Climate model outputs were reprojected to the icosahedral discrete global grid system^55^ and downscaled from 2.50 × 3.75° horizontal resolution to ~100 km resolution using bicubic interpolation. We used the climate anomaly method, so that predicted modelled changes in climate were added (or ratios multiplied for precipitation) to an observed present-day climatology. This method removes any systematic bias from the climate model. The data were then interpolated linearly to 100-year time steps, resulting in 1,201 equal-duration time slices for each of three climatic dimensions used in the simulation. Thus, species responded to climate change on this 100-year temporal scale over the 120-kyr simulation timeframe. Palaeogeography remained constant in the simulations during the 120-kyr simulation (that is, sea level did not fluctuate), because the land–sea mask was static in the climate models and the amount of palaeogeographical change was minimal over this time interval.

### Diversification dynamics

In all simulations, species immediately occupied any suitable cell that they encountered via dispersal, and remained there until the cell became unsuitable via climate change. Environmental changes thus modified geographic distributions of suitable cells uniquely for each species depending on its niche dimensions; species tracked suitable cells through these climatic changes as a function of their dispersal ability. One consequence of environmental change was fragmentation of suitable areas, resulting in either newly isolated populations or elimination of all occupied suitable areas. The former resulted in speciation if populations were isolated for a sufficient length of time (see below), whereas the latter resulted in extinction. Simulations also produced (and stored) a complete phylogeny from each individual starting lineage.

Speciation occurred only allopatrically in our simulations as a direct result of geographic isolation. Minimum isolation time for speciation to occur was set arbitrarily at 10 kyr. In nature, of course, speciation may take longer or shorter than 10 kyr, but this duration is not unreasonable based on both palaeontological and neontological data^60–63^. We chose a time to speciation that was proportional to the time steps available in the climate dataset to generate appreciable numbers of speciation events at the scale of climate change steps, based on our previous analyses^22,35^. Similar simulations^64^ found that assumptions regarding time to speciation did not have significant effects on model results.

Extinction occurred when all occupied suitable habitat (that is, grid cells) for a species disappeared and the species was unsuccessful at colonizing new areas. We applied no specific demographic model or inferred minimum population survivorship threshold, such as might derive from the inclusion of Allee effects. A strict extinction criterion was used because it invoked the fewest assumptions, and because the relatively coarse spatial resolution of the simulation (~7,774 km^2^ per grid) was probably broad with respect to individual life histories.

In all, 1,000 initial species locations were chosen randomly (Fig. 2a) and tested under all combinations of niche breadth (two: narrow and broad), dispersal ability (two: poor and good) and evolutionary scenario (ten; see below), resulting in 40,000 unique simulated species simulations (we subsequently removed 130 seeds due to computational constraints, leaving 870). Each simulated species started at a single grid cell (that is, distributional range of one cell): at the end of each simulation, the final spatial pattern of diversity was quantified, including all extant descendant species that had evolved over the course of the simulation.

### Niche evolution scenarios

We formulated seven broad mechanisms of niche evolution, with varying rates of change, for a total of ten scenarios in our simulations (Fig. 3a, and Supplementary Figs. 1 and 2). Climatic niche evolution occurs over the course of a simulated species’ lifetime. Daughter species are initially bestowed the same niche parameters as their parents, which then evolve independently. Because niche differentiation is not uniform across all dimensions of ecological niches^65^, we allowed the temperature and precipitation niche axes to evolve independently in all evolutionary scenarios.

#### Niche conservatism

This scenario is one in which niches are absolutely conserved in terms of position and breadth. No change occurs in niche traits through time or between daughter and parent lineages.

The next three scenarios are adaptive scenarios, in which niche position and breadth change based on changes in the climatic characteristics within distributional areas between the previous time step and the present time step. That is, if the mean of a given environmental variable across the distribution at time *A* is *V*_*A*_, and the mean in the previous time step is *V*_*A*−1_, then Δ*V*_*A*_ = *V*_*A*_ − *V*_*A*−1_.

#### Directional niche shift

This niche evolution scenario, characterized by a change rate of 10% or 50%, responds to environmental change and is reflective of niche adaptation owing to climate change. Average change in climate (temperature or precipitation niche axes are calculated individually) between two time steps is calculated within the distributional range of a species, and this average change is multiplied by either 0.1 (low rate of change, 10%) or 0.5 (high rate of change, 50%), which is Δ*V*_*A*_. In this scenario, Δ*V*_*A*_ is added to the lower and upper limit of the niche axis (temperature or precipitation). The breadth of tolerances (niche breadth) remains the same in this scenario.

#### Directional niche expansion

In this scenario, the breadth of tolerances can change. When Δ*V*_*A*_ is positive, the upper limit of a species’ tolerance will increase. When Δ*V*_*A*_ is negative (that is, it gets colder or drier), the lower limit of the species’ niche will get colder (temperature) or more drought tolerant (precipitation). Thus, in this scenario and for any given niche axis (temperature or precipitation), only one niche dimension (that is, the upper or lower limit) will change in a time step. We considered slow (10%) and fast (50%) rates of change for Δ*V*_*A*_. Thus, directional niche expansion was one in which, if Δ*V*_*A*_ > 0, the upper niche limit was augmented by Δ*V*_*A*_ × *x*% (where *x* is 0.5 or 0.1, representing 50% and 10% change, respectively) and the lower niche limit was kept constant, and vice versa if Δ*V*_*A*_ < 0.

#### Omnidirectional niche expansion

Each niche axis (temperature or precipitation) changes in both upper and lower tolerance limits. If Δ*V*_*A*_ > 0, the upper tolerance limit will increase and the lower tolerance limit will decrease. Conversely, if Δ*V*_*A*_ < 0, the upper tolerance limit will also increase and the lower tolerance limit will decrease by the absolute value of Δ*V*_*A*_. We considered both slow (10%) and fast (50%) rates of change for Δ*V*_*A*_. Thus, omnidirectional niche expansion was one in which, if Δ*V*_*A*_ > 0, the upper niche limit will be augmented by Δ*V*_*A*_ × *x*% (where *x* is 0.1 or 0.5, representing 10% and 50% change, respectively) and the lower niche limit reduced by the same value, and vice versa if Δ*V*_*A*_ < 0.

In the next three scenarios, we used two random values (Δ*V*_centre*A*_ and Δ*V*_edge*A*_) to control niche dimensions. Δ*V*_centre*A*_ and Δ*V*_edge*A*_ represent (for each of two environmental variables, temperature and precipitation) random numbers between −1*x*NB_0_ and the initial niche breadth (NB_0_), multiplied by 1%. The values could be different in each time step. The niche breadth used was that from the beginning of the simulation for each species.

#### Random niche shift

This scenario is similar to the directional niche shift scenario, but instead of the niche changing based on the climate within the region in which a species lives, the niche changes randomly. For each simulation time step, a random value is selected from −1*x*NB_0_ to NB_0_, multiplied by 1%. This value was then added to the upper and lower limit of the niche axis (either temperature or precipitation). The breadth of the niche remains the same in this scenario. Thus, the lower and upper niche limits were augmented by Δ*V*_centre*A*_, such that the centre changed by Δ*V*_centre*A*_ and the niche breadth remained constant.

#### Random niche expansion or reduction

This scenario institutes random niche change by either expanding both the lower and upper tolerance limits, or by shrinking both the lower and upper tolerance limits. The scenario is similar to the omnidirectional niche expansion scenario, but unlike omnidirectional niche expansion, here the niche can contract. A random number is drawn from −1*x*NB_0_ to NB_0_, multiplied by 1% (Δ*V*_edge*A*_), and this value is added to both the lower and upper niche limit (either temperature or precipitation). Thus, the lower niche limit was augmented by Δ*V*_edge*A*_ and the upper niche limit reduced by Δ*V*_edge*A*_.

#### Random niche change and shift

In this scenario, the niche position and the niche limits change. This niche scenario is a combination of the random niche expansion/reduction and random niche shift scenarios. Two random numbers were used to change both the position of the niche (as in random niche shift) and the limits of the niche (as in random niche expansion/reduction). Thus, the upper niche limit was augmented by Δ*V*_centre*A*_ + Δ*V*_edge*A*_ and the lower niche limit was augmented by Δ*V*_centre*A*_ and then reduced by Δ*V*_edge*A*_.

The same process was applied independently for both the temperature and precipitation niche axes, such that they could evolve at different rates. In the directional niche shift, directional niche expansion and omnidirectional niche expansion scenarios, we instituted both strong (*x* is 0.5 or 50% change) and moderate (*x* is 0.1 or 10%) change in the climatic niche; the slower rate of change is in view of theoretical and empirical results suggesting that distributional change is easier than evolutionary adaptation to new niche conditions^25,31,49,50^.

### Latitudinal diversity gradients

For each niche evolution scenario, we analysed the degree to which our simulations mimicked empirical diversity patterns, such as the latitudinal diversity gradient we analysed in detail in ref. ^22^. Ten initial seeds were selected per 5° latitudinal band across 100 bootstrap replicates, generating mean richness with confidence intervals. The resulting pattern of biodiversity from selected seeds at the end of the simulations was used to construct latitudinal gradients across all dispersal and niche breadth combinations. Simulated latitudinal diversity gradients were compared with empirical diversity gradients for birds and mammals. Distributional data for mammals were derived from the International Union for Conservation of Nature^66^ (accessed 23 January 2023) and for birds from BirdLife International^67^ (accessed 23 January 2023). We intersected distributional range polygons with the hexagonal/pentagonal cells of the spherical geodesic grids, and counted the number of species present in each cell; these values were used to generate latitudinal diversity gradients for 5° latitudinal bands following our previous work^22^.

### Comparing evolutionary dynamics

We calculated speciation rate, extinction rate and net per capita diversification rate using a fixed window approach, with a window size of 1 kyr. Evolutionary metrics were calculated from 10 ka (allowing time for speciation to initiate) to the present day. The evolutionary metrics from the windows were used to test whether the niche conservatism scenario generated higher evolutionary rates using analysis of variance (ANOVA) and post-hoc Tukey’s HSD tests. We ran tests for all niche breadth and dispersal combinations individually and in combination, correcting for multiple comparisons.

In addition to ANOVA, we used paired *t*-tests based on the initial seed cell to compare evolutionary rates between the niche conservatism scenario and all other niche evolution scenarios. The data used in the paired *t*-tests were reduced compared with the data used in the ANOVA, because we ensured all niche breadth × dispersal × window combinations were present across the ten evolutionary scenarios (that is, eliminating scenarios that may have gone extinct in one evolutionary scenario but not in another). Tests were run across all niche breadth × dispersal combinations.

The ANOVA and paired *t*-tests assume independence of the data, which is not necessarily valid when evolutionary rates are measured across multiple time bins for the same ‘clades’, as we did here. We used multiple windows to calculate evolutionary rates, because rates tend to vary with climate change through time (Supplementary Fig. 3). Simple statistical approaches were used to illustrate differences across our evolutionary scenarios, while noting we are able to assess all data due to our simulation framework, and the raw data show the same patterns (presented in Supplementary Figs. 4 and 9).

### Population fragmentation

We examined whether the niche conservatism scenario resulted in more fragmented populations in each year of the simulation, compared with the niche evolution scenarios. A population was defined as an occupied region of suitable habitat that was contiguous and isolated from other such occupied suitable patches. We used an ANOVA and post-hoc Tukey’s HSD test to compare population fragmentation at each seed between the niche conservatism scenario and the other evolutionary niche scenarios, correcting for multiple comparisons as above.

### Reporting summary

Further information on research design is available in the Nature Portfolio Reporting Summary linked to this article.

### Supplementary information

Supplementary Information Supplementary Figs. 1–9 and Tables 1–4.

Reporting Summary

## Data Availability

All data used in the analysis are accessible at 10.6084/m9.figshare.24954597.v4.

## Code availability

All code used to run the simulation are accessible at https://github.com/qiaohj/ees_cpp.
