# Supplementary material for: Ecological niche conservatism spurs diversification in response to climate change
Source: Nat Ecol Evol. 2024 Feb 19;8(4):729–38. doi: 10.1038/s41559-024-02344-5 (PMC11009114; doi:10.1038/s41559-024-02344-5)
Supplement: Supplementary file 1 — Supplementary Figs. 1–9 and Tables 1–4. [file 41559_2024_2344_MOESM1_ESM.pdf]

nature ecology & evolution

Article https://doi.org/10.1038/s41559-024-02344-5

Ecological niche conservatism spurs diversification in response to climate change

In the format provided by the authors and unedited

Supplementary Materials for

Ecological niche conservatism spurs diversification in response to climate change

Huijie Qiao[1*], A. Townsend Peterson[2], Corinne E. Myers[3], Qinmin Yang[4], Erin E. Saupe[5*]

*Corresponding authors: qiaohj@ioz.ac.cn; erin.saupe@earth.ox.ac.uk

The PDF file includes:

Supplementary Figures 1 to 9
Supplementary Tables 1 to 4

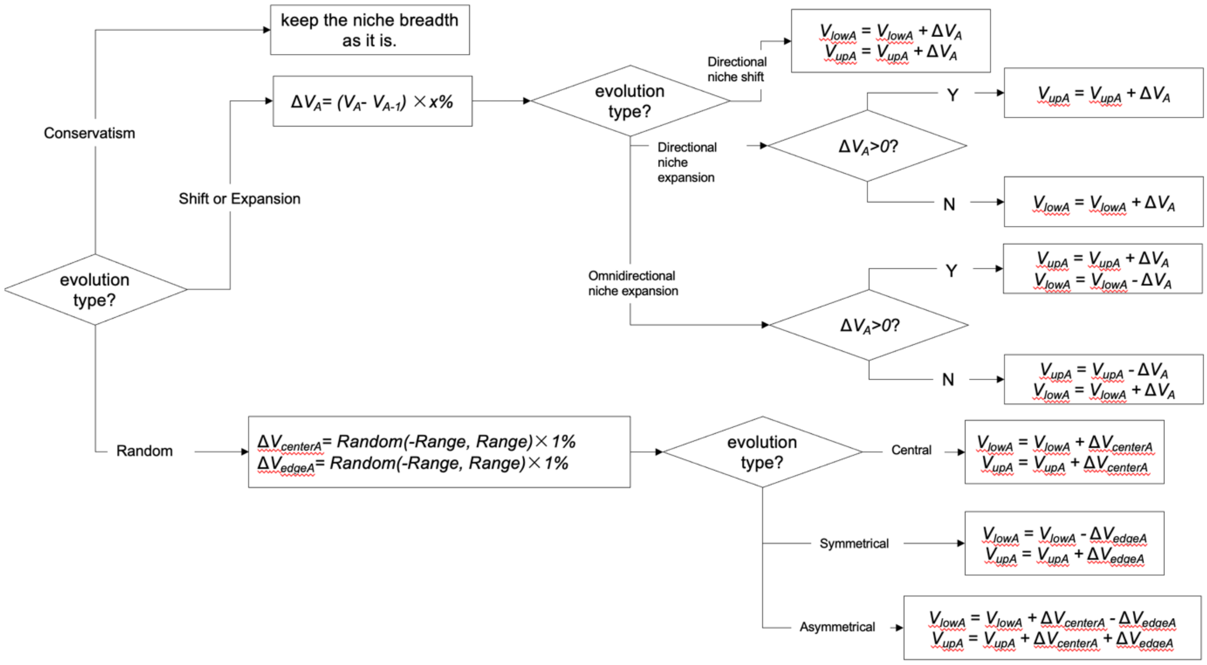

Supplementary Figure 1. Mathematical formulae defining the niche position and niche breadth for the ten niche evolution scenarios used in our simulations: niche conservatism, directional niche shift, directional niche expansion, omnidirectional niche expansion, random niche shift (central), random niche expansion/reduction (symmetrical), and random niche change and shift (asymmetrical).

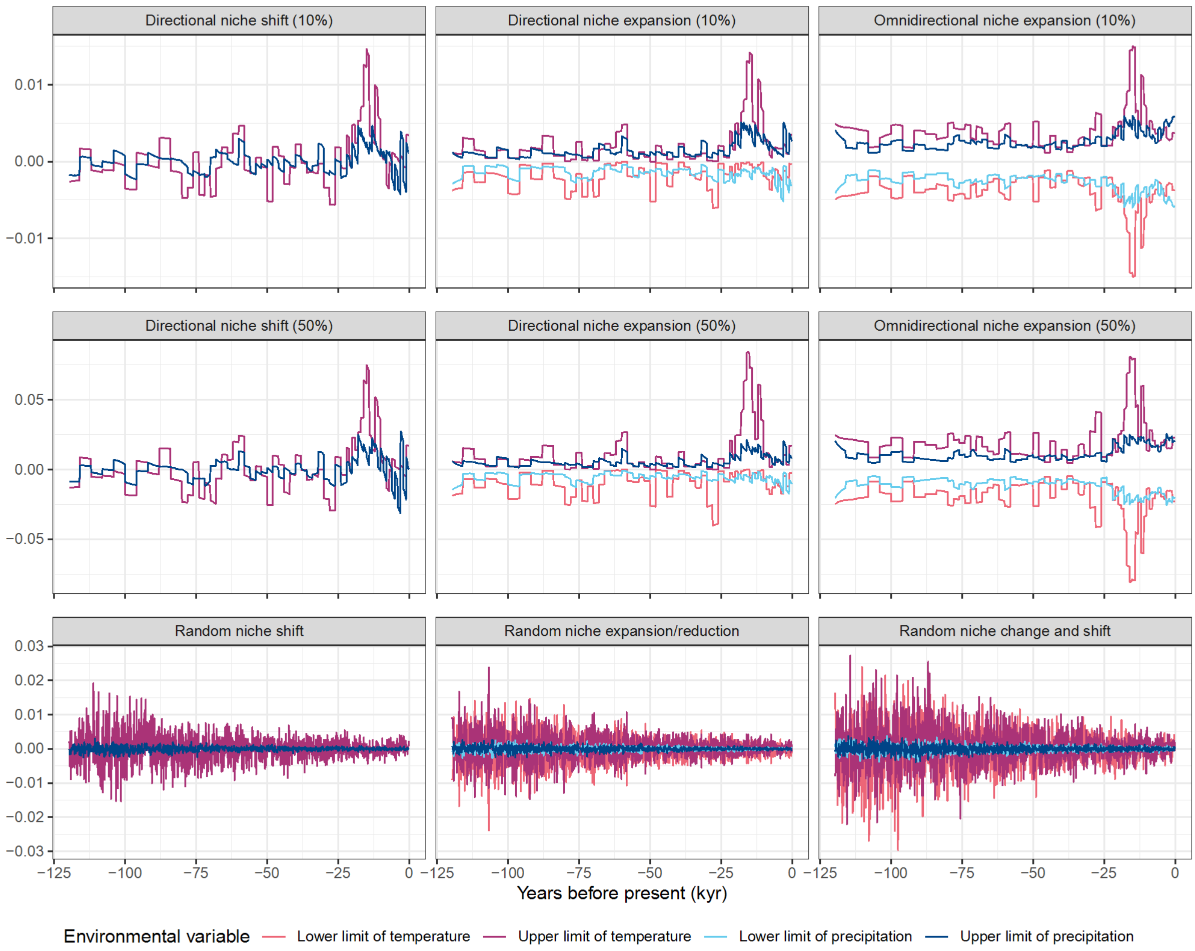

Supplementary Figure 2. Average change in the upper and lower niche axes across all species at a given time step (window) for each niche evolution scenario. The upper limits are shown in dark colours (red for temperature, blue for precipitation) and lower limits are in light colours. The upper and lower limits change in concert for the niche shift scenarios (first column), so only two curves are visible. The random scenarios exhibit reduced variance towards the present day as more species are present over time.

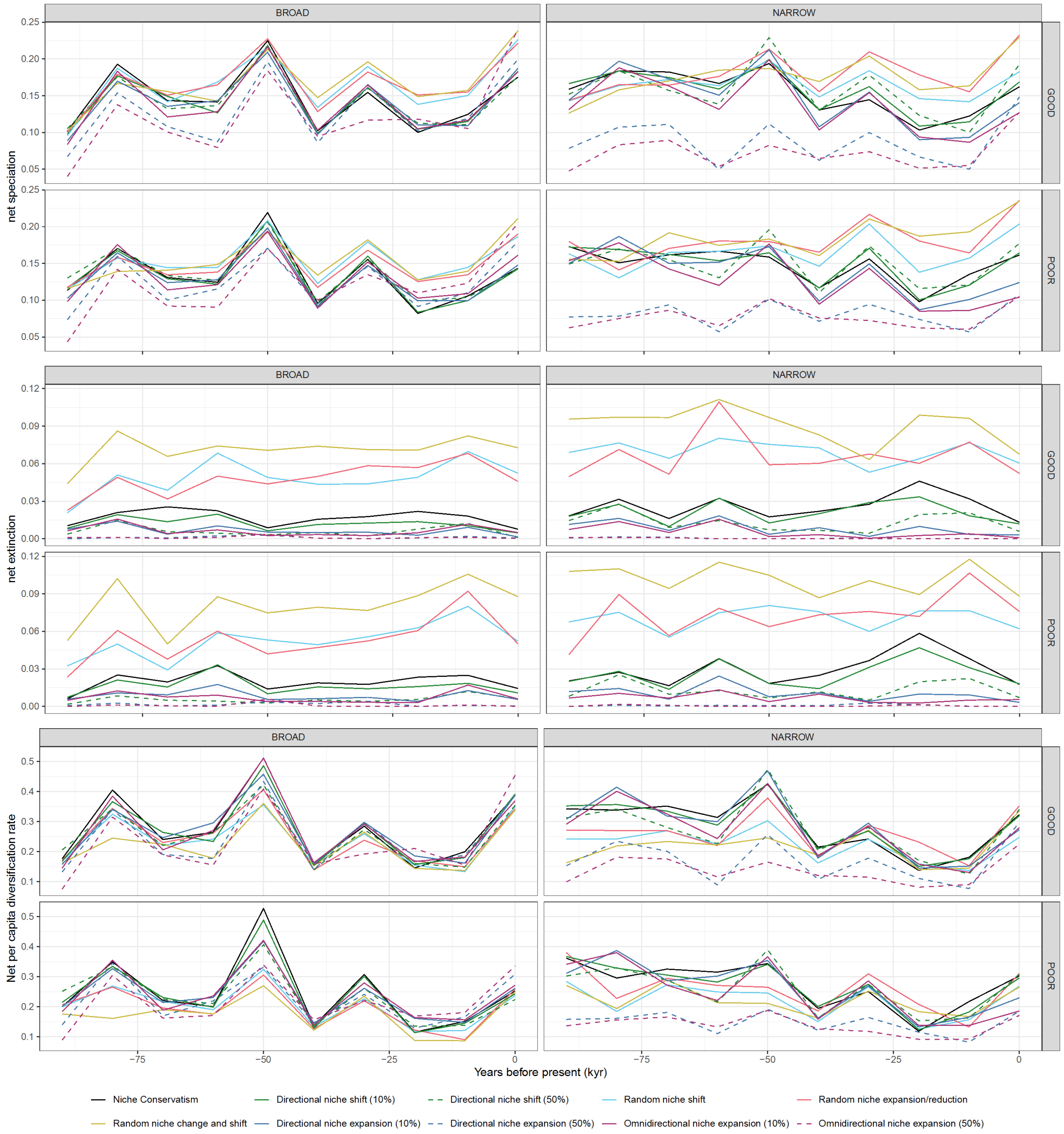

Supplementary Figure 3. Net per capita diversification dynamics during the simulation for each niche evolution, dispersal ability, and niche breadth combination.

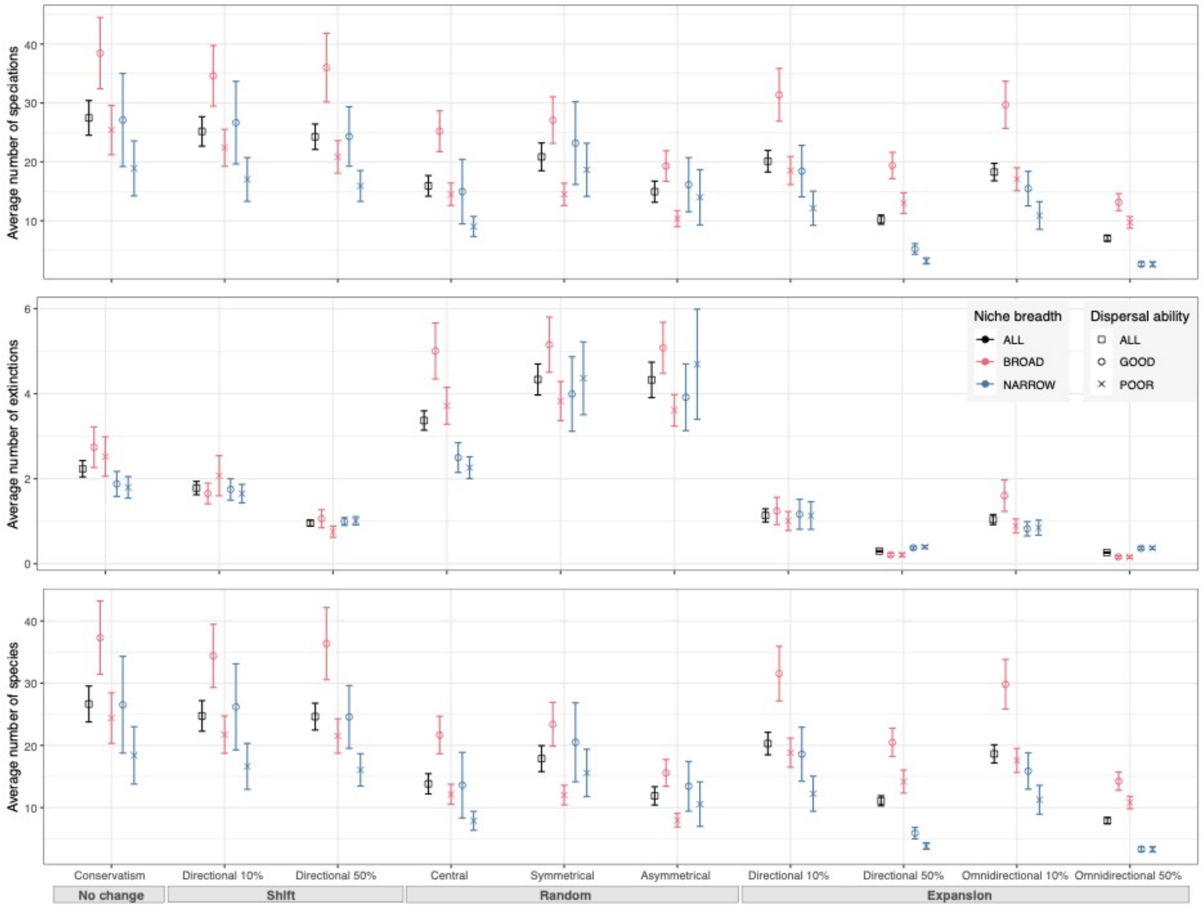

Supplementary Figure 4. Average number of species, speciation events, and extinction events for each niche evolution scenario (n=870 seeds). The bars represent the 95% confidence intervals, as the standard deviations are large and make visualisation difficult. The 'all' category indicates the average number across dispersal and niche breadth combinations (n=3,480 seed by niche breadth by dispersal combinations).

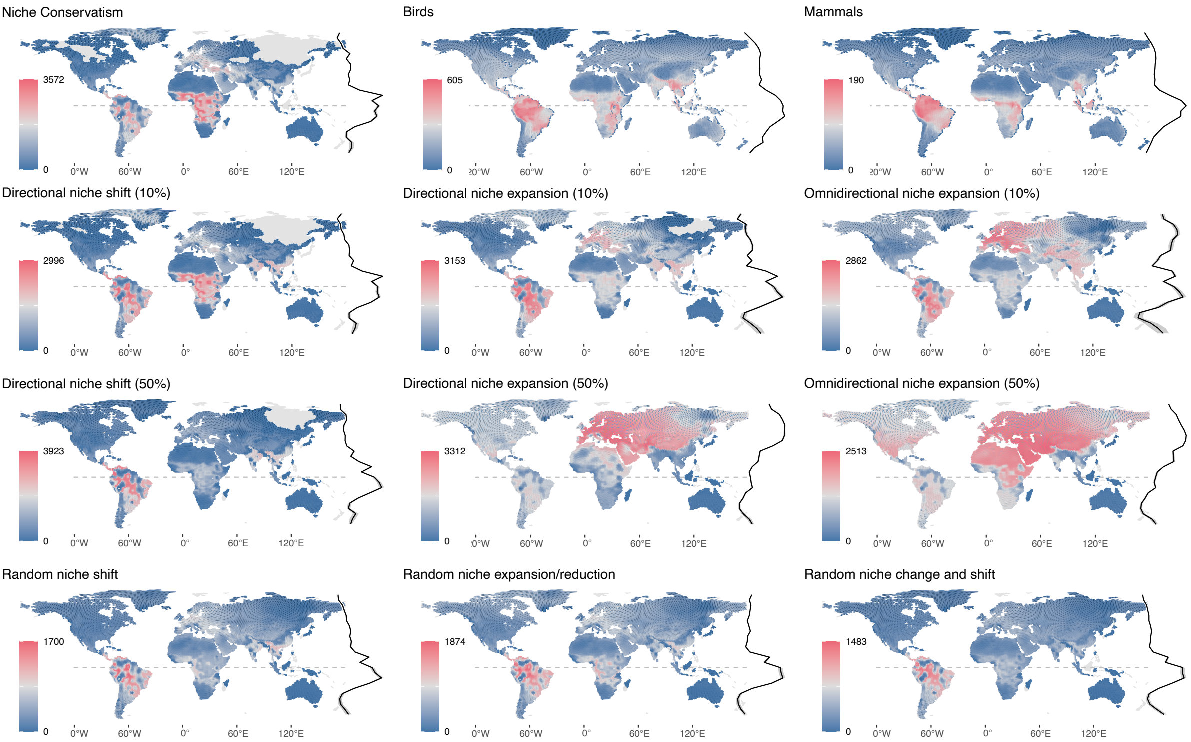

Fig. S5. Latitudinal diversity gradients (LDGs) and patterns of richness at the end of the simulation across all niche breadth and dispersal combinations for each niche evolution scenario. LDGs and richness maps are compared to empirical patterns for mammals and birds derived from the IUCN[66] and from BirdLife International[67], respectively. Map polygons are from the rnaturalearth R package[68].

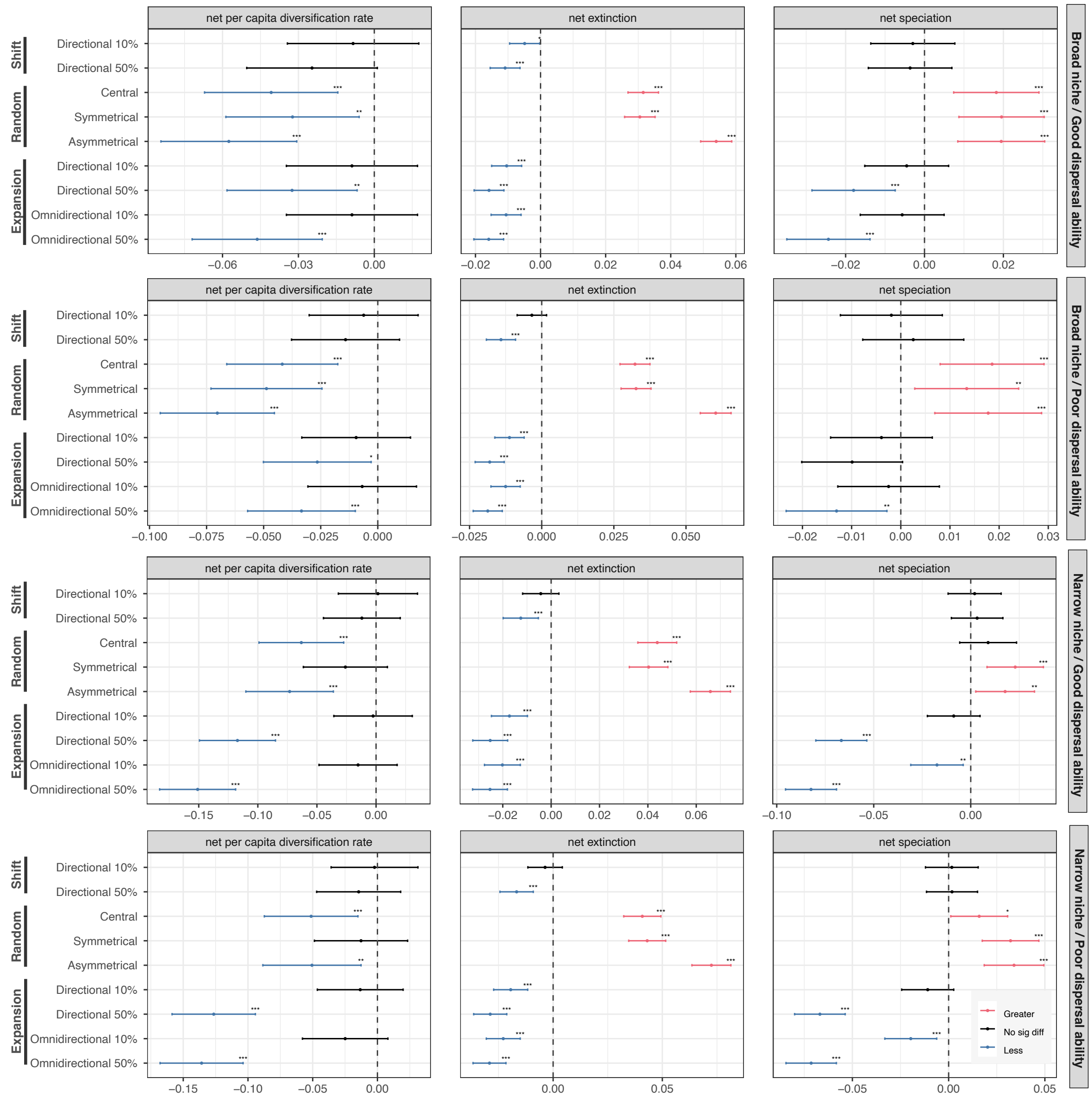

Supplementary Figure 6. Results from post-hoc Tukey's HSD, testing whether the niche conservatism scenario had statistically different net per capita diversification, net per capita extinction, and net per capita speciation rates compared to the other niche evolution scenarios. The 95% confidence interval shows the range of possible values for the difference in means between the niche conservatism scenario and other niche evolution scenarios. Blue indicates that the evolutionary scenarios had lower rates compared to the niche conservatism scenario. Results are presented for each niche breadth and dispersal combination. For the number of datapoints in each comparison, see Table S4. *** indicate statistical significance at α < 0.001, ** < 0.01, and * < 0.05. Significance levels have been corrected for multiple comparisons using Bonferroni.

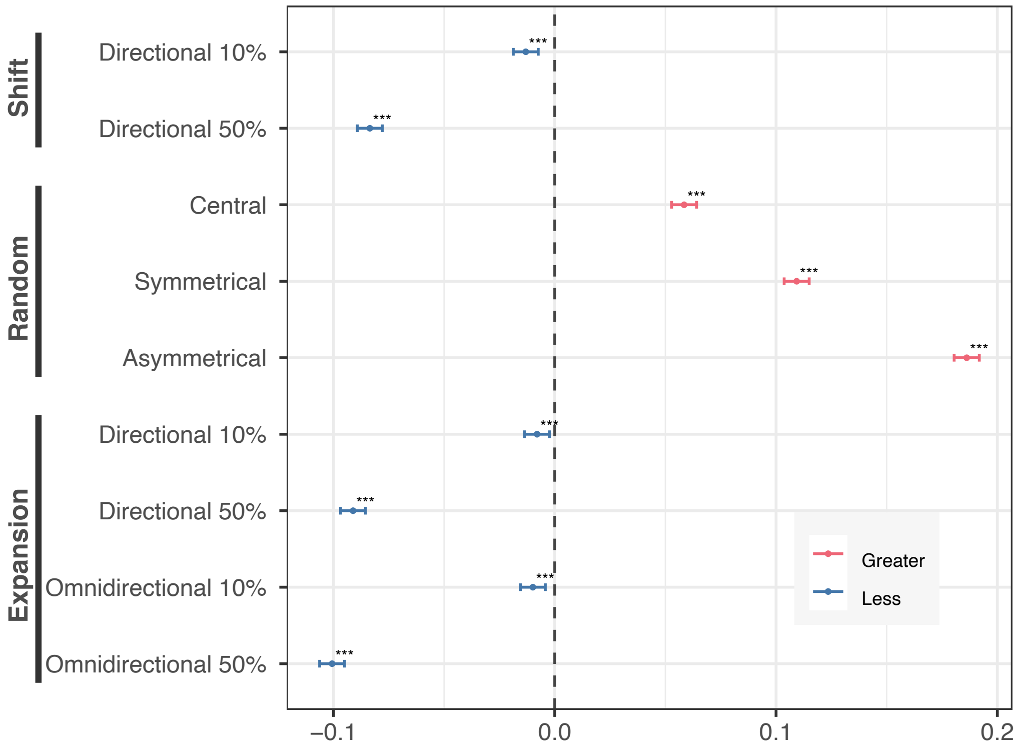

Supplementary Figure 7. Results from post-hoc Tukey's HSD, testing whether the niche conservatism scenario had statistically different population fragmentation compared to the other niche evolution scenarios. The 95% confidence interval shows the range of possible values for the difference in mean fragmentation (central tendency) between the niche conservatism scenario and other niche evolution scenarios. Blue indicates lower fragmentation in the niche evolution scenarios compared to the niche conservatism scenario. Results are presented across all niche breadth and dispersal combinations (n=3,480 seed by niche breadth by dispersal combinations). *** indicate statistical significance at α < 0.001, ** < 0.01, and * < 0.05.

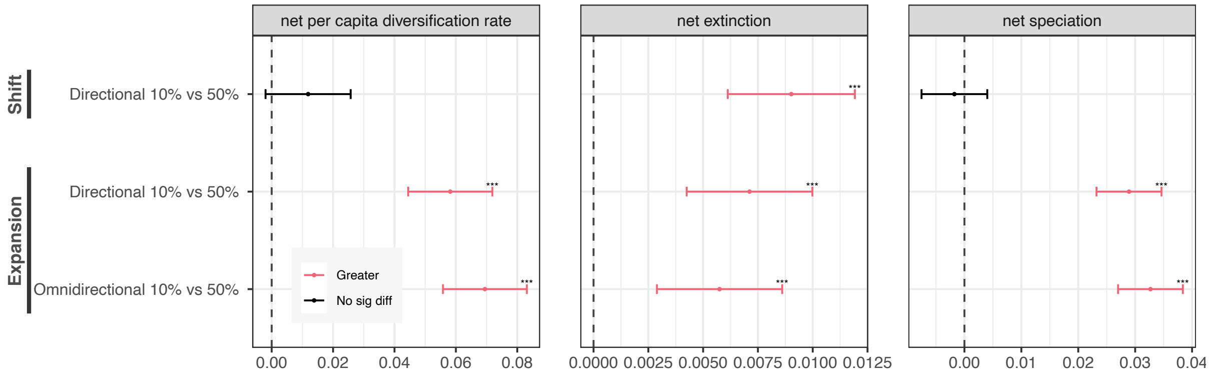

Supplementary Figure 8. Results from post-hoc Tukey's HSD, testing whether evolutionary change scenarios with slower rates had statistically different evolutionary metrics compared to the faster rate of change for that evolutionary scenario. The 95% confidence interval shows the range of possible values for the difference in means (central tendency) between the slow and fast rates of niche change. Red indicates the slow rate of niche change had higher diversification rates compared to the fast rate of niche change for that evolutionary scenario. *** indicate statistical significance at $\alpha < 0.001$, ** < 0.01, and * < 0.05. The number of data points included in each comparison are: 24,692 for directional niche shift 10%; 26,477 for directional niche shift 50%; 25,233 for directional niche expansion 10%; 27,100 directional niche expansion 50%; 25,421 for omnidirectional niche expansion 10%; 27,230 for omnidirectional niche expansion 50%.

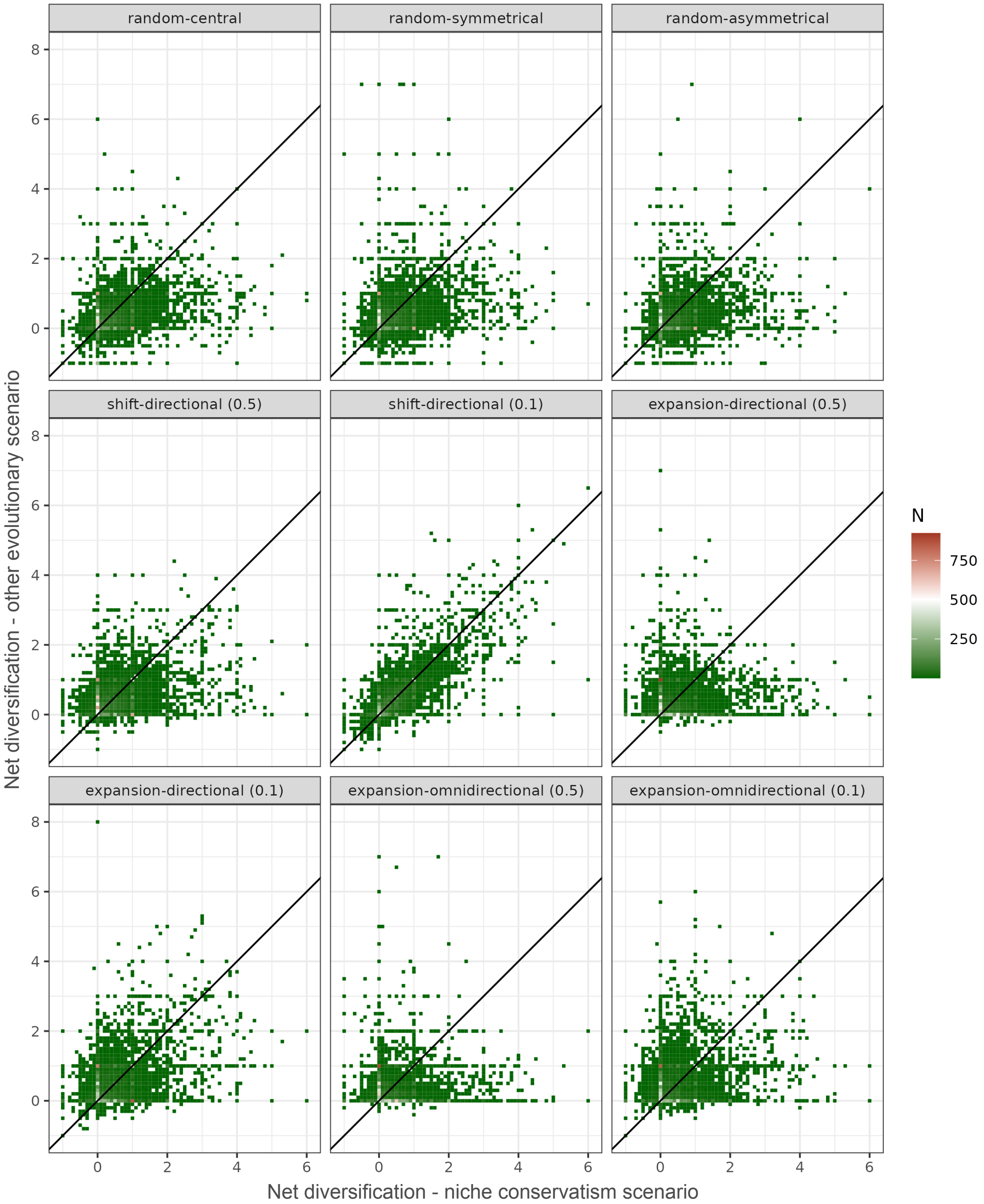

Supplementary Figure 9. Paired net diversification rates for each unique niche breadth x dispersal x time bin (window) combination for the niche conservatism scenario (x-axis) compared to the other evolutionary scenarios (y-axis). These data were used as the basis for the paired *t*-tests in Table S3. Colours represent the number of data points for a particular net diversification value. The black line represents a 1:1 relationship.

Supplementary Table 1. Values used to calculate the number of grid cells searched in a time step. For each time step and occupied grid cell, a number was drawn randomly from 0 to 1. A species could search the number of cells encompassed by this randomly drawn value. Thus, if a value of 0.3102 was drawn for a given time step and grid cell, dispersal would not occur for either poor or good dispersers. Note that poor dispersers could search only two grid cells at maximum within a given time step.

# of cells	0	1	2	3	4
Poor dispersers	0–0.7107	0.7108–0.9996	0.9997–1.0000	-	-
Good dispersers	0–0.3766	0.3767–0.7242	0.7243–0.9766	0.9767–0.9995	0.99996–1.0000

Supplementary Table 2. The mean number of occupied grid cells for each species at the end of the simulation, with interquartile ranges. Distributional ranges of simulated species are provided for each evolutionary niche change scenario and are compared to data for mammals and birds.

**Evolutionary niche scenario**	**25th quartile**	**Mean # of cells**	**75th quartile**
Niche Conservatism	126	486	769
Directional niche shift (50%)	95	488	722
Directional niche shift (10%)	118	475	702
Directional niche expansion (50%)	403	2683	3915
Directional niche expansion (10%)	283	841	1221
Omnidirectional niche expansion (50%)	135	3583	5644
Omnidirectional niche expansion (10%)	267	1135	1611
Random niche shift	72	533	654
Random niche expansion/reduction	54	473	571
Random niche change and shift	46	476	541
Birds	14	371	369
Mammals	8	202	182

Supplementary Table 3. Results from the paired sample *t*-tests, comparing the niche conservatism scenario to all other evolutionary scenarios. Data are paired by seed cell, dispersal ability, niche breadth, and time bin combination (n=16,838). Significance values have been corrected for multiple comparisons using Bonferroni. The 'difference' column indicates whether rates for the niche evolution scenario are significantly less/greater/or not different compared to the niche conservatism scenario. P-values < 0.001 are zero to four significant digits.

**Diversification metric**	**Niche evolution scenario**	p-value	Difference
extinction rate	Directional niche shift (10%)	< .001***	less
extinction rate	Directional niche shift (50%)	< .001***	less
extinction rate	Random niche shift	< .001***	greater
extinction rate	Random niche expansion/reduction	< .001***	greater
extinction rate	Random niche change and shift	< .001***	greater
extinction rate	Directional niche expansion (10%)	< .001***	less
extinction rate	Directional niche expansion (50%)	< .001***	less
extinction rate	Omnidirectional niche expansion (10%)	< .001***	less
extinction rate	Omnidirectional niche expansion (50%)	< .001***	less
net per capita diversification rate	Directional niche shift (10%)	1	no sig. diff
net per capita diversification rate	Directional niche shift (50%)	.006**	less
net per capita diversification rate	Random niche shift	< .001***	less
net per capita diversification rate	Random niche expansion/reduction	< .001***	less
net per capita diversification rate	Random niche change and shift	< .001***	less
net per capita diversification rate	Directional niche expansion (10%)	< .001***	less
net per capita diversification rate	Directional niche expansion (50%)	< .001***	less
net per capita diversification rate	Omnidirectional niche expansion (10%)	< .001***	less
net per capita diversification rate	Omnidirectional niche expansion (50%)	< .001***	less
speciation rate	Directional niche shift (10%)	1	no sig. diff
speciation rate	Directional niche shift (50%)	1	no sig. diff
speciation rate	Random niche shift	< .001***	greater
speciation rate	Random niche expansion/reduction	< .001***	greater
speciation rate	Random niche change and shift	< .001***	greater
speciation rate	Directional niche expansion (10%)	< .001***	less
speciation rate	Directional niche expansion (50%)	< .001***	less
speciation rate	Omnidirectional niche expansion (10%)	< .001***	less
speciation rate	Omnidirectional niche expansion (50%)	< .001***	less

Supplementary Table 4. The number of data points used to test whether the niche conservatism scenario had statistically different net per capita diversification, net per capita extinction, and net per capita speciation rates compared to the other niche evolution scenarios in Fig. S6.

Evolutionary scenario		Broad niche breadth		Narrow niche breadth	
		Good dispersal	Poor dispersal	Good dispersal	Poor dispersal
No change	Conservatism	7657	7525	4540	4455
Shift	Directional 10%	7742	7619	4727	4604
	Directional 50%	7943	7889	5367	5278
Random	Central	7316	7067	3642	3457
	Symmetrical	7313	7072	3754	3514
	Asymmetrical	6802	6370	3246	2935
Expansion	Directional 10%	7813	7687	4901	4832
	Directional 50%	8050	7970	5600	5480
	Omnidirectional 10%	7812	7719	4977	4913
	Omnidirectional 50%	8060	7980	5620	5570
