# Supplementary material for: Ecological niche conservatism spurs diversification in response to climate change
Source: Nat Ecol Evol. 2024 Feb 19;8(4):729–38. doi: 10.1038/s41559-024-02344-5 (PMC11009114; doi:10.1038/s41559-024-02344-5)
Supplement: Supplementary file 2 — Reporting Summary [file 41559_2024_2344_MOESM2_ESM.pdf]

nature portfolio

Corresponding author(s): Huijie Qiao & Erin E Saupe

Last updated by author(s): Jan 6, 2024

Reporting Summary

Nature Portfolio wishes to improve the reproducibility of the work that we publish. This form provides structure for consistency and transparency in reporting. For further information on Nature Portfolio policies, see our Editorial Policies and the Editorial Policy Checklist.

Statistics

For all statistical analyses, confirm that the following items are present in the figure legend, table legend, main text, or Methods section.

n/a	Confirmed	
☐	☒	The exact sample size (*n*) for each experimental group/condition, given as a discrete number and unit of measurement
☐	☒	A statement on whether measurements were taken from distinct samples or whether the same sample was measured repeatedly
☐	☒	The statistical test(s) used AND whether they are one- or two-sided<br>*Only common tests should be described solely by name; describe more complex techniques in the Methods section.*
☐	☒	A description of all covariates tested
☐	☒	A description of any assumptions or corrections, such as tests of normality and adjustment for multiple comparisons
☐	☒	A full description of the statistical parameters including central tendency (e.g. means) or other basic estimates (e.g. regression coefficient) AND variation (e.g. standard deviation) or associated estimates of uncertainty (e.g. confidence intervals)
☐	☒	For null hypothesis testing, the test statistic (e.g. *F*, *t*, *r*) with confidence intervals, effect sizes, degrees of freedom and *P* value noted<br>*Give P values as exact values whenever suitable.*
☒	☐	For Bayesian analysis, information on the choice of priors and Markov chain Monte Carlo settings
☒	☐	For hierarchical and complex designs, identification of the appropriate level for tests and full reporting of outcomes
☒	☐	Estimates of effect sizes (e.g. Cohen's *d*, Pearson's *r*), indicating how they were calculated

Our web collection on statistics for biologists contains articles on many of the points above.

Software and code

Policy information about availability of computer code

Data collection	This study relied on simulations with virtual species and thus most data were generated in silico. The empirical data were derived from the IUCN for mammals (accessed 1/23/2023) and from BirdLife International for birds (accessed 1/23/2023), which are publicly available.
Data analysis	All code used in the analysis is accessible here: https://github.com/qiaohj/ees_cpp.

For manuscripts utilizing custom algorithms or software that are central to the research but not yet described in published literature, software must be made available to editors and reviewers. We strongly encourage code deposition in a community repository (e.g. GitHub). See the Nature Portfolio guidelines for submitting code & software for further information.

Data

Policy information about availability of data

All manuscripts must include a data availability statement. This statement should provide the following information, where applicable:

- Accession codes, unique identifiers, or web links for publicly available datasets
- A description of any restrictions on data availability
- For clinical datasets or third party data, please ensure that the statement adheres to our policy

All data, code, and materials used in the analysis will be made available and are currently accessible here: https://github.com/qiaohj/ees_cpp.

Research involving human participants, their data, or biological material

Policy information about studies with human participants or human data. See also policy information about sex, gender (identity/presentation), and sexual orientation and race, ethnicity and racism.

Reporting on sex and gender	N/A
Reporting on race, ethnicity, or other socially relevant groupings	N/A
Population characteristics	N/A
Recruitment	N/A
Ethics oversight	N/A

Note that full information on the approval of the study protocol must also be provided in the manuscript.

Field-specific reporting

Please select the one below that is the best fit for your research. If you are not sure, read the appropriate sections before making your selection.

☐ Life sciences ☐ Behavioural & social sciences ☒ Ecological, evolutionary & environmental sciences

For a reference copy of the document with all sections, see nature.com/documents/nr-reporting-summary-flat.pdf

Ecological, evolutionary & environmental sciences study design

All studies must disclose on these points even when the disclosure is negative.

Study description	We use a spatially explicit mechanistic model to examine the relationship between climate niche evolution and net diversification rate for terrestrial virtual species. We decompose the relative contributions of speciation and extinction to net diversification under varying degrees of climate niche evolution, allowing a direct test of the two opposing hypotheses.
Research sample	The simulations relied on virtual species. Patterns of speciation and extinction were simulated in response to a dynamic climate approximated over the last 120,000 years, derived from the coupled atmosphere-ocean generalized circulation models of HadCM3. The virtual species could interact with spatiotemporal variation in temperature and precipitation by shifting their distributions or climatic niches, during which process they could undergo speciation or extinction. We chose 1,000 initial virtual species locations (seed cells) randomly, which were tested under all combinations of niche breadth (narrow and broad), dispersal ability (poor and good), and niche evolution scenario, resulting in 40,000 unique virtual species simulations.
Sampling strategy	We chose 1,000 initial virtual species locations (seed cells) randomly.
Data collection	No data were required for the simulations, but we compared empirical distributional patterns for birds and mammals to our simulated patterns. The empirical data derived from the IUCN for mammals (accessed 1/23/2023) and from BirdLife International for birds (accessed 1/23/2023), which are publicly available.
Timing and spatial scale	Simulations were run over the last 120,000 years using realistic estimates for how temperature and precipitation changed spatially over this time interval. Climate data were derived from the HadCM3 coupled atmosphere-ocean generalized circulation model. The spatial resolution of the simulations was 1 degree.
Data exclusions	We removed 5,200 simulations (130 seed cells) prior to analysis, because these simulations were characterized by run-away speciation, resulting in run times too long (>100 hours) or requiring too much memory (>500 GB).
Reproducibility	We repeated our simulations at each of 1000 seed cells to assess variability of simulated patterns globally.
Randomization	N/A
Blinding	N/A

Did the study involve field work? ☐ Yes ☒ No

Reporting for specific materials, systems and methods

We require information from authors about some types of materials, experimental systems and methods used in many studies. Here, indicate whether each material, system or method listed is relevant to your study. If you are not sure if a list item applies to your research, read the appropriate section before selecting a response.

Materials & experimental systems

n/a	Involved in the study
☒	☐ Antibodies
☒	☐ Eukaryotic cell lines
☒	☐ Palaeontology and archaeology
☒	☐ Animals and other organisms
☒	☐ Clinical data
☒	☐ Dual use research of concern
☒	☐ Plants

Methods

n/a	Involved in the study
☒	☐ ChIP-seq
☒	☐ Flow cytometry
☒	☐ MRI-based neuroimaging
